# Combination of Zinc and All-Trans Retinoic Acid Promotes Protection against *Listeria monocytogenes* Infection

**DOI:** 10.1371/journal.pone.0137463

**Published:** 2015-09-09

**Authors:** Yussaira Castillo, Masato Tachibana, Yukiko Nakatsu, Kenta Watanabe, Takashi Shimizu, Masahisa Watarai

**Affiliations:** The United Graduate School of Veterinary Science, Yamaguchi University, 1677–1 Yoshida, Yamaguchi 753–8515, Japan; INRA Clermont-Ferrand Research Center, FRANCE

## Abstract

Zinc (Zn) is the second most abundant transition metal after iron. It plays a vital role in living organisms and affects multiple aspects of the immune system. All-trans retinoic acid (atRA) is an isomeric form of the vitamin A or retinol. It possesses the greatest biological activity of Vitamin A. Vitamin A and related retinoids influence many aspects of immunity. In this study, we demonstrated that treatment with a combination of Zn and atRA contributes to host resistance against infection by *Listeria monocytogenes*. Pretreatment with Zn and atRA enhanced resistance against *L*. *monocytogenes* infection in mice and treatment with both Zn and atRA showed a higher protective effect than treatment with either alone. Supplementation with Zn, atRA or their combination decreased the number of *L*. *monocytogenes* present in target organs. In vitro, supplementation increased the bacterial uptake by macrophage cells and reduced the replication of *L*. *monocytogenes*. Our results suggest that the combination of Zn and atRA has a great bacteriostatic impact on *L*. *monocytogenes* and its infection.

## Introduction

One promising area of current public health research is the possibility that micronutrient supplementation may decrease the incidence of infectious disease. With the development of modern medicine, the prevention, treatment, and cure of infectious diseases have become progressively dependent on vaccines and refined antimicrobial drugs. For decades, we have focused on the production of newer and stronger medicines and have failed to remember that simple compounds can effectively contribute to the treatment or prevention of infectious diseases. Among the micronutrients, zinc (Zn) and vitamin A are thought to have the largest impact on the prevention of diseases. Their deficiencies have been known to increase susceptibility to infection and to raise the incidence of infectious respiratory and alimentary tract diseases [1, 2].

Zn is well known to be both an essential and toxic micronutrient for development of all organisms, including bacteria [3, 4]. Zn is the second most abundant transition metal in the human body and has crucial roles in many facets influencing growth and affecting the development and integrity of the immune system. It is important for enzymes of all six classes, as well as transcription and replication factors [5]. Studies reveal that supplementation and optimal intake of Zn restore impaired immune responses and decrease the incidence of infection in vivo. T-cells levels increase significantly after Zn supplementation and cell-mediated immune response are improved [6, 7]. The in vivo and in vitro effects of Zn on immune cells depend mainly on the Zn concentration, considering the fact that Zn has significant toxicity at high concentrations. However, the molecular basis of Zn toxicity remains poorly defined [8–10].

All-trans retinoic acid (atRA), also known as tretinoin, is the acid form of vitamin A. This appears to be its active form in all tissues except retina [11]. Supplementation with vitamin A and its metabolite, atRA, has been reported to decrease the incidence and severity of infectious diseases, although the regulation of immune function by vitamin A may also vary widely depending on the type of infection and the immune responses involved [12]. Certain aspects of a functional synergy between Zn and vitamin A are well defined. Zn status influences several aspects of vitamin A metabolism, including: 1) absorption–Zn is essential for the lymphatic absorption of retinol; 2) transport–Zn is fundamental for the synthesis of retinol-binding protein; and 3) utilization–Zn is required for the conversion of retinol to retinal for dark adaptation. There is also evidence that vitamin A affects Zn absorption and utilization [13, 14].

Listeriosis is caused by the Gram-positive bacterium *Listeria monocytogenes*. In humans, this pathogen has the ability to cross the intestinal, placental, and blood-brain barriers, leading to gastroenteritis, materno-fetal infections, and meningoencephalitis, respectively. A key feature of the virulence of *L*. *monocytogenes* is its ability to avoid the killing mechanisms of professional and non-professional phagocytic host cells [15, 16]. *L*. *monocytogenes* infections in humans are caused mainly by the ingestion of contaminated foods, such as dairy products, raw vegetables, fish, poultry, processed chicken, and beef [17].

Worldwide, few trials of dietary supplements have examined the practical importance of the Zn-vitamin A interaction for human respiratory tract affections and diarrhea [18–20]. Those that have been conducted have suggested that Zn and vitamin A statuses directly affect the morbidity of these infections in their trial populations. Since both Zn and vitamin A primarily affect aspects of innate immunity, the effect of supplementation against infection with facultative intracellular pathogen *L*. *monocytogenes* was examined in mice.

## Materials and Methods

### Bacterial strain

*L*. *monocytogenes* EGD was maintained as a frozen glycerol stock and cultured in brain heart infusion (BHI) broth (Becton Dickinson, Franklin Lakes, NJ, USA) or on BHI broth containing 1.5% agar.

### Zn or atRA treatment in mice

**Fig 1:**
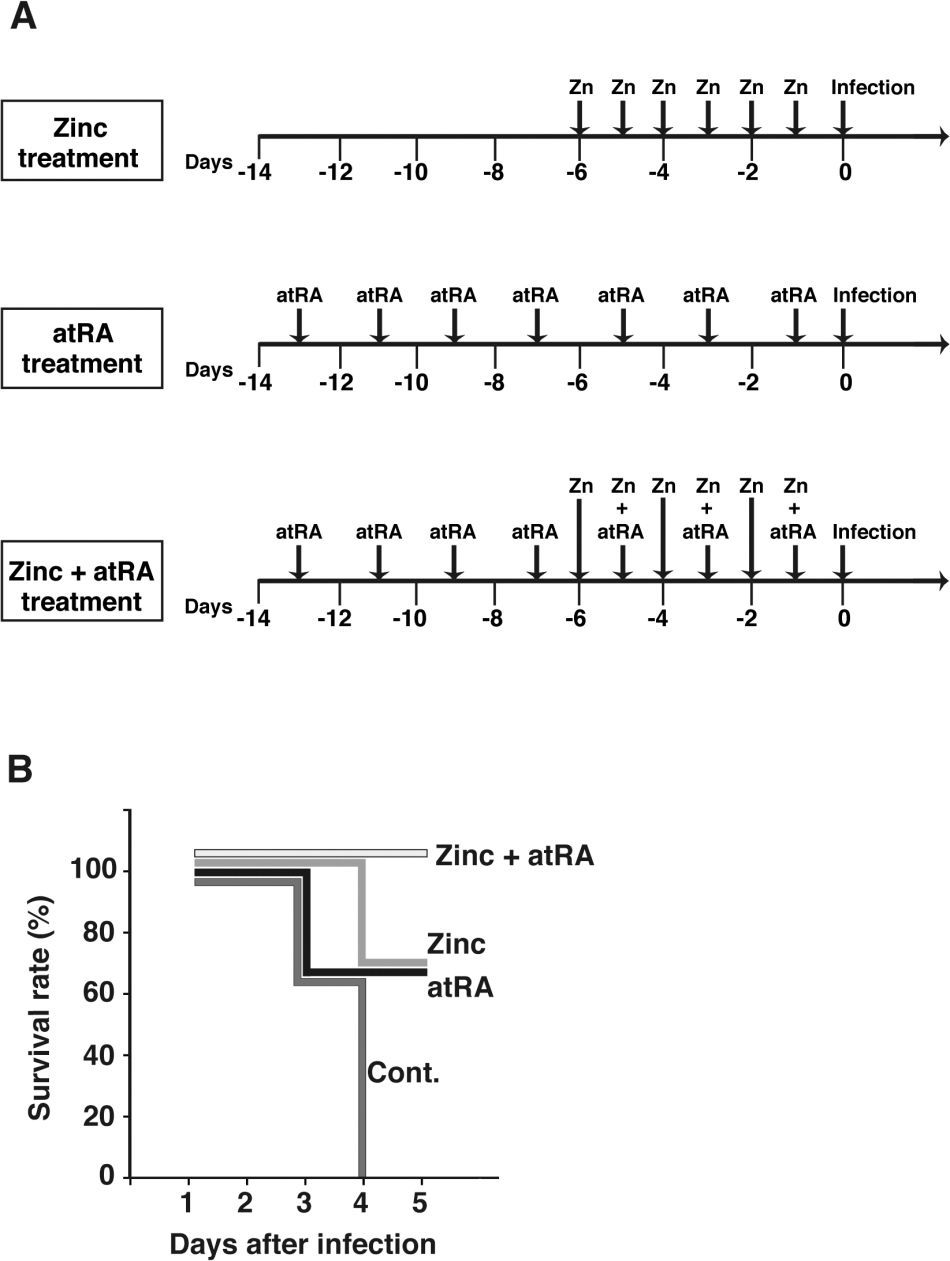
Survival of lethally infected mice supplemented with Zn and atRA. Panel A shows the administration procedure. At the end of atRA and Zn administration, mice were inoculated intravenously with 10^5^/100 μl *L*. *monocytogenes* per animal. Groups of four mice per compound were checked daily for survival. Survival was observed until day 5 post-infection.

Six to 10-week-old BALB/c male mice were obtained from Kyudo Co., Ltd. (Saga, Japan). All mice were maintained under specific pathogen-free conditions in sterile cages which were put into a ventilated isolator. Fluorescent lights were cycled 12 hours on/12 hours off, and ambient temperature (23±1°C) and relative humidity (40–60%) were regulated. Groups of four mice per assay were used. Mice were intraperitoneally administered 150 μg of ZnSO_4_ (Wako, Osaka, Japan) in 0.2 ml PBS or 400 μg of atRA (Wako) dissolved in 0.2 ml corn oil. PBS or corn oil was injected, as a control. ZnSO_4_ was administered six times over 1 week; atRA was administered seven times over 2 weeks (Fig 1A). Pre-treated mice were infected intravenously via the tail vein with approximately 10^5^ cells of *L*. *monocytogenes* in 0.1 ml saline and survival was observed on day 5 of infection. Depending on the progress of the disease, animals were monitored every 3–4 hours during the day-phase (7:00 am to 7:00 pm) by veterinarian. Mice were determined endpoints when mice showed loss of ability to ambulate and inability to access food or water, and mice were sacrificed by isoflurane euthanasia. Humane endpoint by isoflurane euthanasia was conducted if death of the animals during the following hours was to be expected. For counting the number of bacteria in the liver and spleen, pre-treated mice were infected intravenously with approximately 10^4^ cells of *L*. *monocytogenes* in 0.1 ml saline. Two days after infection, their liver and spleen were removed and homogenized in saline. The tissue homogenates were serially diluted with PBS and plated on BHI agar plates to estimate the number of colony-forming units (CFU). All the protocols for animal experiments have been approved in the Animal Research Committee of Yamaguchi University (Permit Number: 141). Animal studies were performed in compliance with the Yamaguchi University Animal Care and Use guidelines. The mice were sacrificed by cardiac puncture under isoflurane anesthesia and overdose of isoflurane, and all efforts were made to minimize suffering by using isoflurane anesthesia.

### Cytokine measurement

The serum levels of IFN-γ, TNF-α, IL-6, and IL-1ß were measured for infected and uninfected mice. Pre-treated mice were infected intravenously with approximately 10^4^ CFU of *L*. *monocytogenes* in 0.1 ml saline and blood was collected at 2 days after infection. Blood was collected at the same times for uninfected mice. Serum levels of cytokines were measured with an enzyme linked immunosorbent assay (ELISA) kit (Bio legends, San Diego, CA, USA), according to the manufacturer’s instructions.

### Cell culture

J774 cells were cultured in RPMI 1640 (Sigma, St. Louis, MO, USA) containing 10% FBS. J774 cells were seeded (1–2 × 10^5^ per well) in 12-well tissue culture plates.

### Efficiency of phagocytosis and bacterial survival

J774 cells were grown for 3–4 days in 12-well tissue culture plates to confluency. ZnSO_4_ (40 μM) or atRA (20 μM) was added to the J774 cells 48 h before infection. Bacterial strains were deposited onto J774 cells at a multiplicity of infection of 0.1 by centrifugation at 150 × g for 10 min at room temperature. To measure phagocytosis efficiency, after 30 min of incubation at 37°C, the cells were washed once with medium and then incubated in a medium containing gentamicin (50 μg/ml, Sigma) for 30 min. The cells were then washed three times with PBS and lysed with cold distilled water. CFU values were determined by serial dilution on BHI plates. To measure bacterial survival efficiency, the infected macrophages were incubated at 37°C for 30 min, washed once with RPMI 1640, and incubated with RPMI 1640 containing gentamicin (50 μg/ml) for 24 h. Cell washing, lysis, and plating procedures were the same as for the bacterial invasion efficiency assay. Cell viability analysis was the use of trypan blue dye exclusion staining.

### *In vitro* bacterial growth assay

Bacterial strains were cultured in BHI broth containing ZnSO_4_ (200 or 1,000 μM) or atRA (10 or 50 μM) at 37°C for 24 h. CFU values were determined by serial dilution on BHI plates.

### Statistical analyses

One-way ANOVA was used to make statistical comparisons between the groups. Results with *p* < 0.05 were considered significantly different and are indicated by asterisks. Data are expressed as the mean of triplicate samples from three identical experiments and the error bars represent the standard deviations (SD).

## Results

### Pretreatment with Zn and atRA enhances host resistance against *L*. *monocytogenes* infection

**Fig 2:**
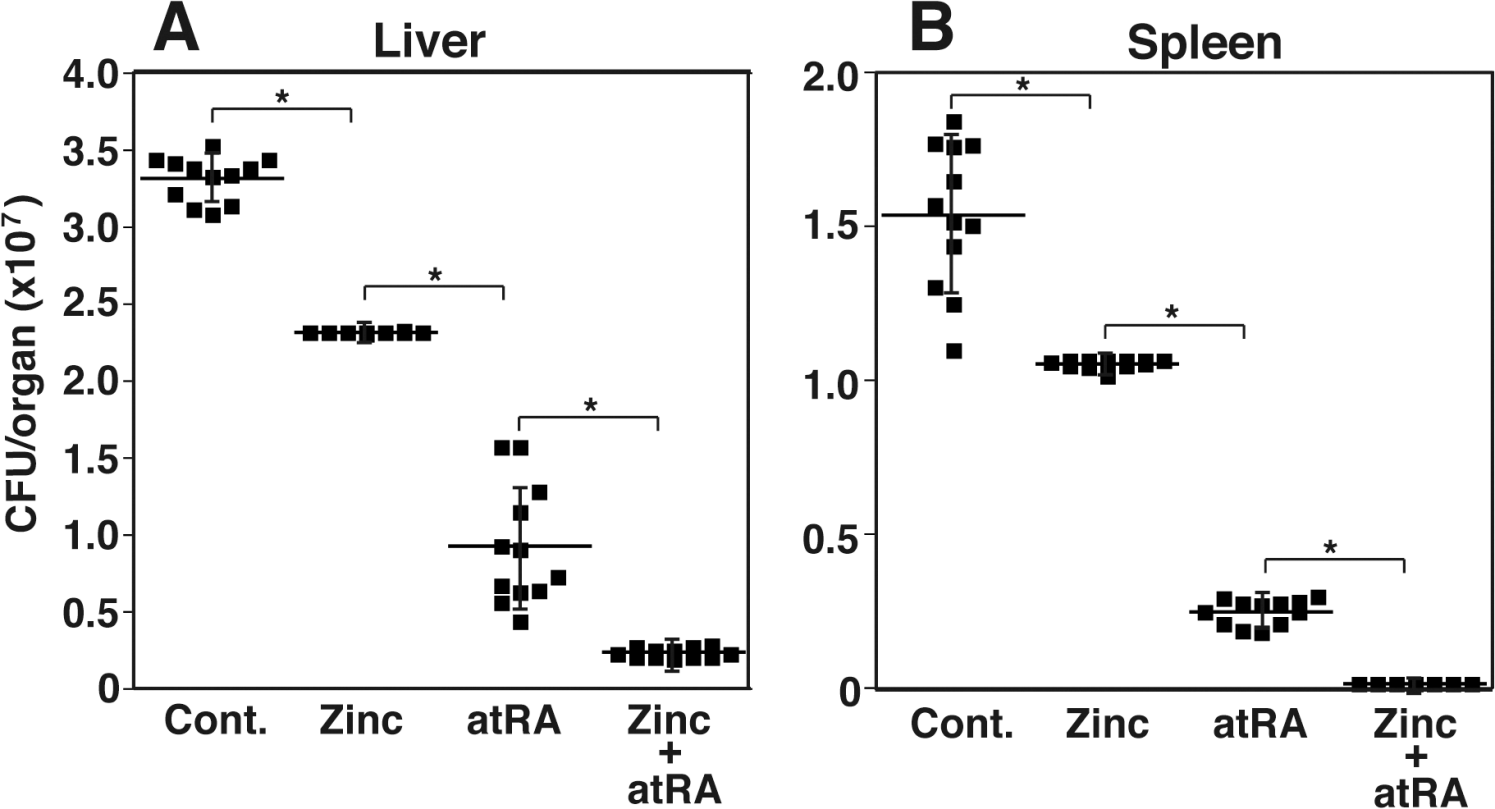
Number of *L*. *monocytogenes* in the target organs of Zn- and atRA-supplemented mice. Mice were injected intraperitoneally with Zn, atRA or a combination of both compounds. Twenty-four hours after the last dose, the mice were infected intravenously with 10^4^/100 μl per mouse. CFU was measured in the liver and spleen 48 h after infection. The experiment was performed three times with four mice per group each time. Control mice received corn oil alone. Data are mean ± SD values of 12 mice per group. Statistically significant differences, compared with each group, are indicated by asterisks.

To examine the effect of Zn and atRA on *L*. *monocytogenes* infection, Zn- or/and atRA-pretreated mice were infected intravenously with *L*. *monocytogenes*. All the control mice died within 4 days (Fig 1B). In contrast, 66.6% of Zn- or atRA-pretreated mice survived at least 5 days (Fig 1B). All of the mice pretreated with both Zn and atRA survived for 5 days (Fig 1B). Next, to measure bacterial number in the liver and spleen, the mice were infected with *L*. *monocytogenes* and the bacterial number were determined 2 days after infection. The number of bacteria observed in these organs was significantly lower in pretreated mice than in control mice (Fig 2). Treatment with the combination of Zn and atRA showed a higher protective effect than treatment with either alone (Fig 2).

### Pretreatment with Zn and atRA affect cytokine production

**Fig 3:**
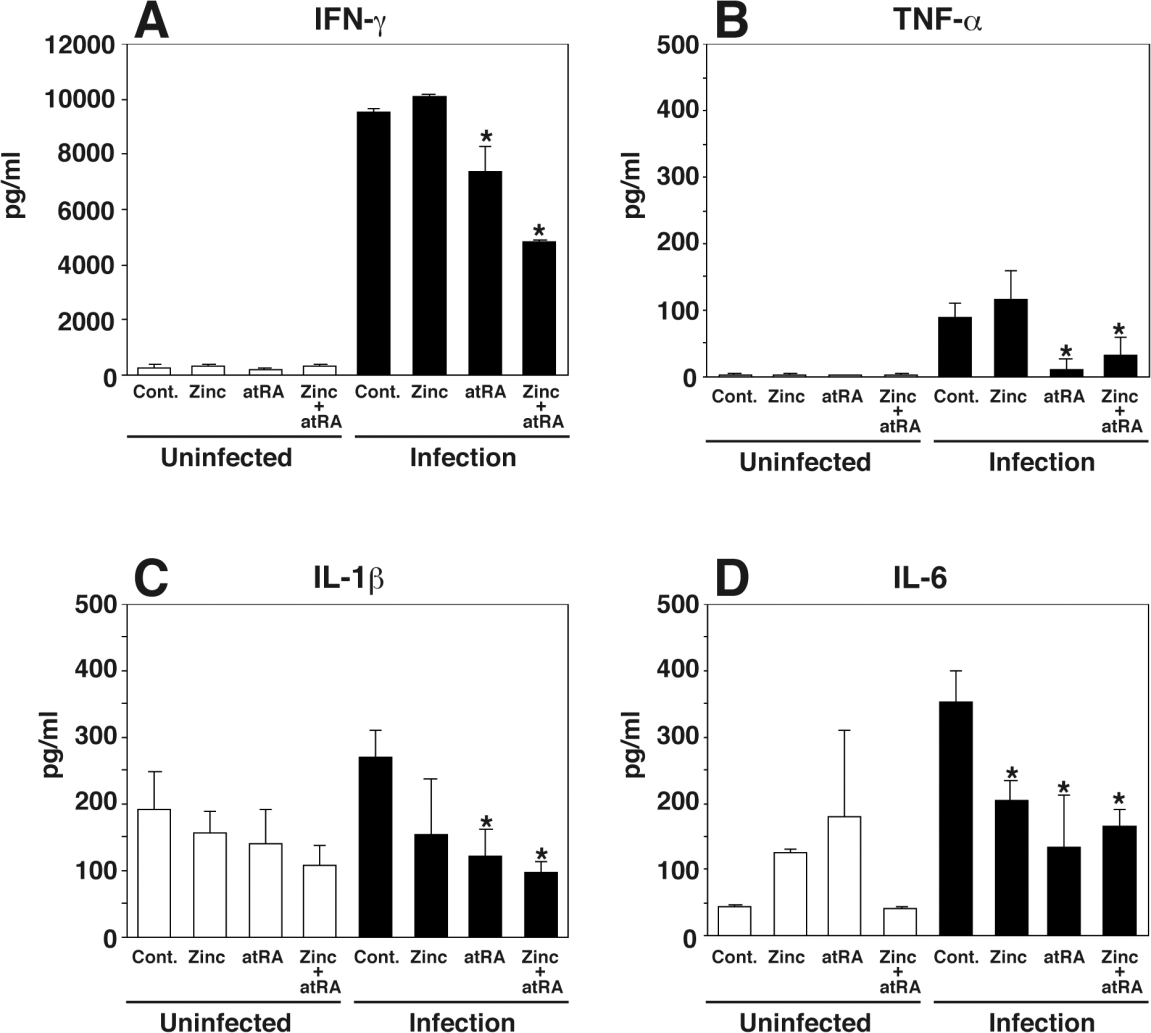
Cytokine levels in normal and *L*. *monocytogenes*-infected mice supplemented with Zn, atRA, or a combination of both. Doses were given intraperitoneally for 7 intradays and 6 consecutive days, respectively (Fig 1A). Twenty-four hours after the last dose, the mice were infected intravenously with 10^4^/100 μl *L*. *monocytogenes* per mouse. IFN-γ, TNF-α, IL-1β, and IL-6 were measured using an ELISA 48 h post-infection. Experiment was performed three times with four mice per group each time. Control mice received corn oil (atRA) or PBS (Zn) alone. Data are mean ± SD values of 12 mice per group. Statistically significant differences, compared with the control, are indicated by asterisks.

Multiple components of the cellular compartment of both the innate and the adaptive immune systems are simultaneously required for the resistance to infection by *L*. *monocytogenes*. To investigate the effect of Zn and atRA pretreatment on cytokine production, pretreated mice were infected with *L*. *monocytogenes* and cytokine production was determined 2 days after infection using ELISA kits. *L*. *monocytogenes* infection induced the production of IFN-γ, TNF-α, IL-1ß, and IL-6 (Fig 3). Pretreatment with atRA or a combination of Zn and atRA decreased IFN-γ, TNF-α, and IL-1ß production (Fig 3A–3C). Pretreatment with Zn, atRA, or a combination of Zn and atRA decreased IL-6 production (Fig 3D).

### Zn and atRA treatment affect bacterial infection in J774 cells

**Fig 4:**
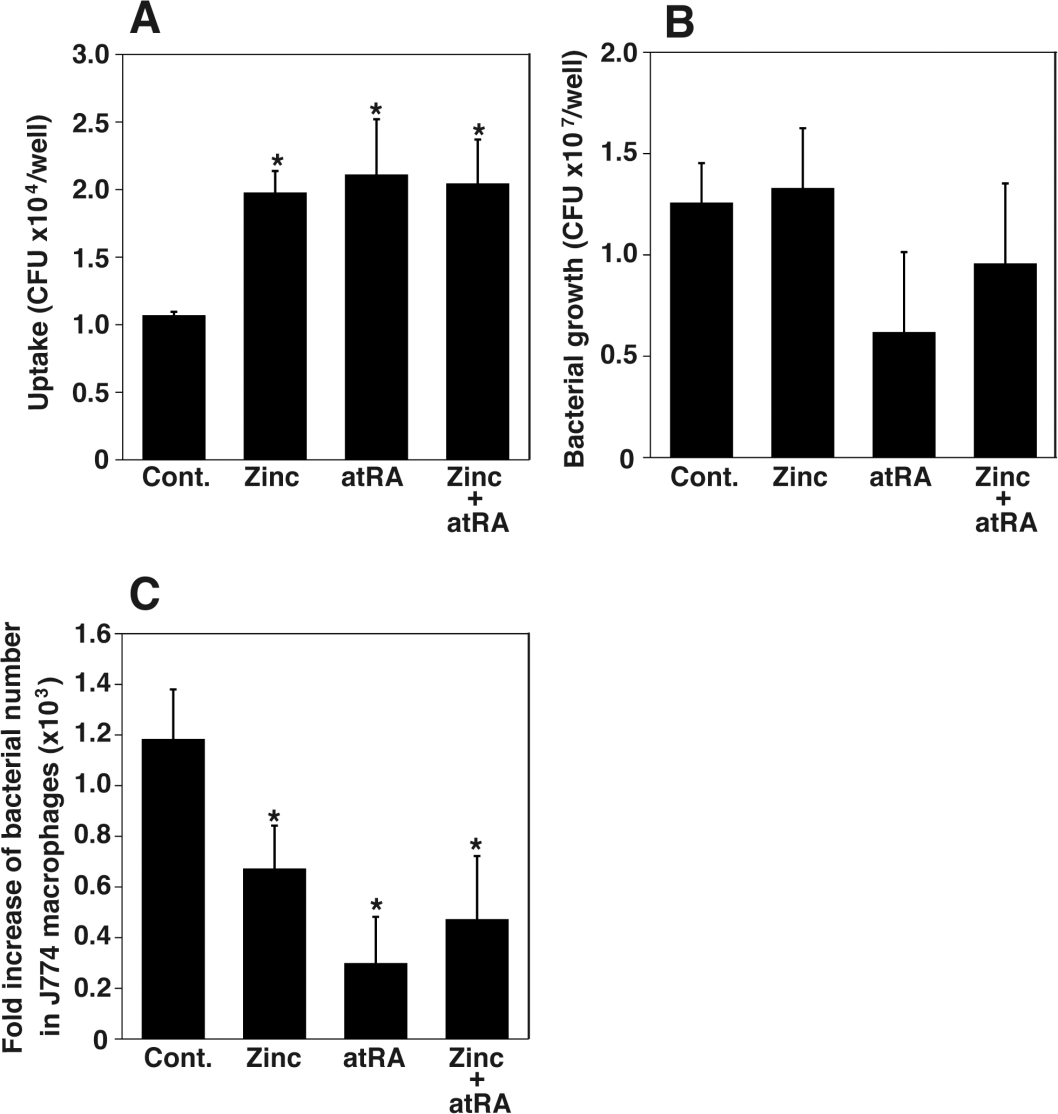
*L*. *monocytogenes* macrophage uptake and bacterial survival after Zn and atRA supplementation. J774 macrophage cells were infected with *L*. *monocytogenes* by centrifugation at 150 × g for 10 min at room temperature at a multiplicity of infection of 0.1. Bacterial uptake (A) was measured after 30 min of incubation at 37°C and bacterial survival (B) was measured after 24 h. ZnSO_4_ (40 μM) or atRA (20 μM) were added 48 h before infection. Panel C shows fold increase of bacterial number in macrophages. Results are presented as fold increase of bacterial number relative to the number of phagocytosed bacteria. Data are the averages of triplicate samples from three identical experiments and error bars represent SD. Statistically significant differences, compared with the control, are indicated by asterisks.

To examine the effect of Zn and atRA on phagocytosis and bacterial survival in J774 cells, J774 cells pretreated with Zn and atRA were infected with bacteria. Since J774 cells were infected with bacteria at low multiplicity of infection, J774 cells still were intact as seen by light microscopy at 24 h after infection. Cell viability was also confirmed by the trypan blue dye exclusion staining. Pretreatment with Zn, atRA, or a combination of Zn and atRA significantly increased the relative phagocytosis efficiencies of *L*. *monocytogenes* in J774 cells (Fig 4A). Pretreatment with these reagents inhibited growth of *L*. *monocytogenes* in J774 cells (Fig 4B and 4C).

### Zn has bacteriostatic effect against *L*. *monocytogenes in vitro*

**Fig 5:**
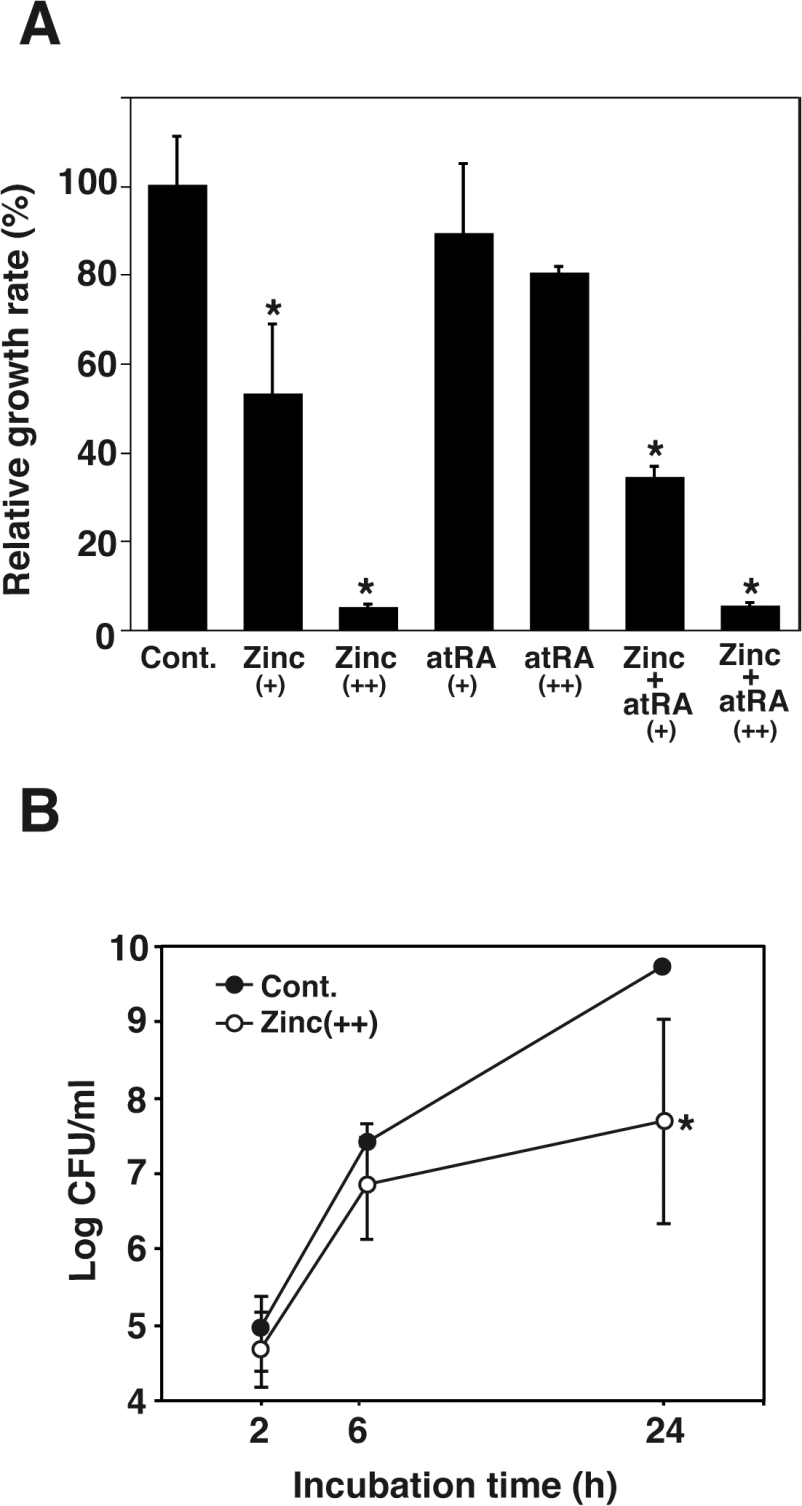
*L*. *monocytogenes* growth in vitro in the presence of Zn, atRA or their combination. *L*. *monocytogenes* was cultured in BHI broth for 24 h in tubes containing ZnSO_4_ (+; 200 or ++; 1,000 μM) or atRA (+; 10 or ++; 50 μM) at 37°C. CFU values were determined by serial dilution on BHI plates. For each sample, CFU values of *L*. *monocytogenes* observed in the absence of Zn and atRA was set at 100% and CFU values in the presence of Zn and atRA was showed relative to this value. Data are the averages of triplicate samples from three identical experiments and error bars represent SD. Statistically significant differences, compared with the control, are indicated by asterisks. Panel B shows growth curves of bacteria in BHI broth with or without Zn.

To investigate the bacteriostatic abilities of Zn and atRA, bacteria were cultured in BHI broth with or without Zn and/or atRA. Although the addition of atRA to the growth medium did not affect the growth of *L*. *monocytogenes* (atRA+, 89.4%; atRA++, 80.3%), addition of Zn significantly reduced the growth of *L*. *monocytogenes* in a dose-dependent manner (Zn+, 53.7%; Zn++, 4.8%; Zn and atRA+, 34.5%, Zn and atRA++, 5.9%) (Fig 5A and 5B).

## Discussion

Many biochemical and physiological roles of Zn have been indicated, reinforcing the importance of Zn in various biological phenomena, such as the immune response [21]. It has been reported that the influence of cadmium (Cd) and Zn on mice improves resistance to *L*. *monocytogenes* infection. Cd-exposed mice are more susceptible to *L*. *monocytogenes* infection, while Zn significantly reduces the negative effect of Cd on the antimicrobial defense of mice [22]. Because the effect of Zn treatment alone against *L*. *monocytogenes* infection remained unknown, we investigated the role of Zn in *L*. *monocytogenes* infection in mice in this study. Our results showed that Zn contributes to the defense against *L*. *monocytogenes* infection in mice. The protective ability of Zn appears to be due to a bacteriostatic effect on *L*. *monocytogenes*. Although Zn is an essential micronutrient for bacteria, it is toxic at high concentrations [23]. Zn plays a protective role against infection by other pathogens, such as shiga-toxigenic *Escherichia coli* [24] and *Trypanosoma evansi* [25]. A bacteriostatic effect of Zn was demonstrated using in vitro bacterial growth assays. These results suggest that Zn acts on bacteria directly.

Vitamin A is a fat-soluble essential nutrient that is acquired from the diet as atRA, retinyl esters, or β-carotene. Retinoic acid (RA) can be generated in multiple isoforms although the all-trans isoform predominates in most tissues [26]. Vitamin A or RA is important for an extensive range of biological processes, including immunomodulatory functions [27]. Vitamin A deficiency can lead to an increased susceptibility to infectious diseases [2]. During infection with *Toxoplasma gondii*, an intracellular replicating pathogen controlled by IFN-γ [28], the acute Th1 response, and parasite clearance are significantly impaired in vitamin A-deficient mice [27]. RA has been shown to enhance macrophage activation in response to *Mycobacterium tuberculosis* infection in vitro [29]. Our results also suggest that pretreatment with atRA also enhances host resistance against *L*. *monocytogenes* infection.

Although Zn and atRA have been shown to protect mice from infection by intracellular pathogens in previous studies and in our study, the mechanism is still unclear. In our study, pretreatment with Zn or atRA inhibited bacterial growth in the liver, spleen, and macrophages; and these protective effects were enhanced by pretreatment with a combination of Zn and atRA. These results suggest that the bacteriostatic effect is increased by a combination of Zn and atRA. Experimental and human studies have demonstrated that type 1 cytokines, including IL-1β, IL-6, IFN-γ, and TNF-α, play an important role in the development of cell-mediated immune responses to intracellular infections. Taking this into consideration, we speculated that the bacteriostatic effect increased by the combination of Zn and atRA, is caused by the induction of cytokine production. The production of cytokines in mouse serum after *L*. *monocytogenes* infection was measured using ELISAs. Unexpectedly, Zn and atRA treatment decreased the production of cytokines. The decrease in cytokine production may be caused by the decrease in bacterial numbers in mice due to Zn and atRA treatment and its bacteriostatic effect. Although vitamin A and Zinc supplementation, individually, reduces morbidity and mortality from diarrhoeal and respiratory disease in general, it is unclear whether this compounds enhances immunity against all pathogens or has specific effects for certain organisms. The modulation of immune function by supplementation of combined atRA and Zn appeared to be complex and may involve many arms of the immune system since is unclear how this compounds combines to enhance immunity.

Because the bacteriostatic activity of J774 macrophages was increased by Zn and atRA treatment, Zn and atRA may affect phagocytosis by macrophages. Indeed, it has been reported that the supplementation of Zn 1or atRA enhances bacterial clearance by macrophages and phagocytosis [30, 31]. Although no enhancement of the bacterial clearance activity of macrophages by treatment with a combination of Zn and atRA was observed in vitro, the combination treatment may be effective in vivo or in primary macrophages.

The detailed mechanism by which the combination of Zn and atRA enhances the bacteriostatic effect is still unclear; this is a task for future research. Our results show that treatment with a combination of Zn and atRA may be useful against infection by intracellular pathogens and may provide a new therapeutic option for bacterial infection.
